# Low-normal serum unconjugated bilirubin levels are associated with late but not early carotid atherosclerotic lesions in T2DM subjects

**DOI:** 10.3389/fendo.2022.948338

**Published:** 2022-11-02

**Authors:** Chun-Hua Jin, Jun-Wei Wang, Jiang-Feng Ke, Jing-Bo Li, Mei-Fang Li, Lian-Xi Li

**Affiliations:** ^1^ Department of Endocrinology and Metabolism, Shanghai Sixth People's Hospital Affiliated to Shanghai Jiao Tong University School of Medicine, Shanghai Clinical Medical Center of Diabetes, Shanghai Key Clinical Center of Metabolic Diseases, Shanghai Institute for Diabetes, Shanghai Key Laboratory of Diabetes, Shanghai, China; ^2^ Department of Endocrinology and Metabolism, Shanghai Songjiang District Central Hospital, Songjiang Hospital Affiliated to Shanghai Jiao Tong University School of Medicine, Shanghai, China; ^3^ Department of Cardiology, Shanghai Sixth People's Hospital Affiliated to Shanghai Jiao Tong University School of Medicine, Shanghai, China; ^4^ Department of Emergency, Shanghai Sixth People's Hospital Affiliated to Shanghai Jiao Tong University School of Medicine, Shanghai, China

**Keywords:** serum unconjugated bilirubin, serum bilirubin, carotid intima-media thickness, carotid plaque, carotid stenosis, type 2 diabetes mellitus

## Abstract

**Aims:**

We aimed to examine the association of serum unconjugated bilirubin (UCB) within normal limits with carotid atherosclerosis in Chinese patients with type 2 diabetes mellitus (T2DM).

**Methods:**

This cross-sectional, real-world study was performed in 8,006 hospitalized T2DM patients including 4,153 men and 3,853 women with normal UCB. The subjects were stratified into quintiles based on serum UCB levels (<6.2, 6.2–7.9, 8.0–8.9, 9.0–10.9, and >10.9 μmol/l, respectively). Carotid atherosclerotic lesions detected by ultrasonography, including carotid intima-media thickness (CIMT), carotid plaque, and stenosis, were compared among the five groups. The associations of serum UCB levels and quintiles with carotid atherosclerotic lesions were also determined by multiple logistic regression.

**Results:**

The prevalence of carotid plaque (55.3%, 49.5%, 47.4%, 43.8%, and 37.5%, respectively; *p* < 0.001 for trend) and stenosis (15.2%, 12.2%, 9.1%, 7.7%, and 5.4%, respectively; *p* < 0.001 for trend) was progressively lower across the UCB quintiles even after adjusting for age, sex, and duration of diabetes. Results of a fully adjusted multiple logistic regression analysis revealed that serum UCB levels and quintiles were significantly associated with carotid plaque and stenosis. Compared with the subjects in the lowest UCB quintile, the risk of carotid plaque decreased by 25.5%, 28.7%, 33.5%, and 42.8%, and that of carotid stenosis by 24.6%, 37.4%, 44.9%, and 47.3%, respectively, in those from the second to highest UCB quintiles. High serum UCB within the normal range was a protective factor against carotid plaque [odds ratio (OR) 0.810, 95% confidence interval (CI) 0.747–0.878; *p* < 0.001] and stenosis [OR 0.722, 95% CI 0.647–0.805; *p* < 0.001]. However, no significant association was observed between serum UCB and CIMT in T2DM patients. Furthermore, C-reactive protein (CRP) levels were significantly higher in the subjects with carotid atherosclerosis than in those without carotid atherosclerosis and clearly decreased across the UCB quintiles.

**Conclusions:**

Serum UCB within normal limits is inversely associated with late carotid atherosclerotic lesions including carotid plaque and stenosis but not CIMT, an early carotid atherosclerotic lesion in T2DM patients. High-normal UCB may be protective against carotid atherosclerosis by its anti-inflammation effect, which was indicated by significantly decreased CRP levels from the lowest to highest UCB quintiles.

## Introduction

Bilirubin, one of the final products of heme metabolism and long considered merely as a toxic waste, has been known for its strong antioxidant, anti-inflammatory, and neuroprotective activities in recent decades (1–5). As a result, many studies focused on the associations between serum bilirubin levels and chronic inflammatory diseases such as ischemic arterial diseases, especially coronary heart disease (CHD), but with controversial conclusions (6–10). For example, some studies found that serum bilirubin was negatively related to clinically manifested cardiovascular disease and events (6–8), whereas others discovered a U-shaped correlation between bilirubin levels and the risk of CHD (9, 10). Furthermore, relevant studies conducted in a diabetic population have not been sufficiently investigated.

Recently, several observational studies have demonstrated that serum bilirubin may have a protective effect on diabetic macrovascular complications (11, 12). For example, the study in Korean women with type 2 diabetes mellitus (T2DM) from Kim et al. (11) showed that, as a continuous variable, a 1-SD increment in total bilirubin (TB) levels was associated with a significant 30% reduction in arterial stiffness diagnosed with brachial-ankle pulse wave velocity, and when categorized in tertiles, the risk of arterial stiffness in the highest TB tertile obviously reduced by 51% relative to that in the lowest tertile. Furthermore, the Fenofibrate Intervention and Event Lowering in Diabetes (FIELD) study found that there was a remarkably inverse association between baseline plasma TB concentrations and lower-limb amputation, and, in T2DM subjects, patients with every 5-μmol/l decrement on TB concentrations faced a 38% higher risk of first amputation (12).

However, the abovementioned studies involved in the relationship between bilirubin and atherosclerotic diseases mainly focused on TB levels and did not distinguish conjugated bilirubin (CB) and unconjugated bilirubin (UCB) from TB. TB is composed of CB and UCB. Research showed that UCB, also known as indirect bilirubin (I-BIL), has more effective antioxidant and anti-lipoperoxidative properties than CB, also known as direct bilirubin (D-BIL) (13, 14). In addition, most relevant studies mainly compared bilirubin levels among different groups without considering bilirubin levels above or within the reference range. Therefore, data on the effect of UCB especially within physiological concentrations on atherosclerotic lesions were quite few, and the conclusion remained unclear.

Furthermore, limited information is available to assess the possible relationship of low-grade UCB with early and late carotid atherosclerosis in T2DM patients. Therefore, the aim of our study was to comprehensively investigate the associations between serum UCB within the normal limits and carotid atherosclerotic lesions, including carotid intima-media thickness (CIMT), atherosclerotic plaque, and stenosis in Chinese T2DM patients.

## Materials and methods

### Study population

A total of 11,805 T2DM patients hospitalized in the Endocrinology and Metabolism Department of Shanghai Jiao Tong University Affiliated Sixth People’s Hospital during the period from January 2003 to December 2012 were recruited in this cross-sectional, real-world study. The exclusion criteria included 1) patients under 18 years of age; 2) acute diabetic complications such as diabetes ketoacidosis; 3) pregnancy; 4) lack of information on anthropometric, clinical, carotid ultrasound measurements; 5) severe liver diseases other than non-alcoholic fatty liver diseases (e.g., any hepatitis, liver cirrhosis, liver tumor); 6) abnormal serum bilirubin (defined as TB >18 µmol/l and/or CB >6 µmol/l in our hospital); 7) diseases affecting serum bilirubin such as hemochromatosis and obstruction of the bile duct; 8) systemic inflammatory diseases; and 9) progressive malignancy. Ultimately, 8,006 participants joined in this analysis. All subjects signed written consent forms, and the current study was approved by the Ethics Committee of Shanghai Jiao Tong University Affiliated Sixth People’s Hospital (approval number: 2018-KY-018(K)) and adhered to the tenets of the Declaration of Helsinki.

In our hospital, serum TB and CB concentrations were measured using a LABOSPECT 008 AS (Hitachi High-Tech Co., Tokyo, Japan) autoanalyzer in the biochemistry laboratory, but the serum UCB level was not routinely measured. Therefore, the serum UCB level used in the present study was calculated by subtracting the serum CB concentration from the TB concentration as serum TB is the sum of CB and UCB.

Social and medical histories, including smoking, alcohol use, and duration of diabetes (DD), were obtained by interviewing the subjects. Medication history including antiplatelet agents (APAs), antihypertensive agents (AHAs), lipid-lowering drugs (LLDs), and insulin or insulin analogues (IIAs) was also recorded.

### Physical examination and laboratory measurements

The subjects underwent measurements of systolic blood pressure (SBP), diastolic blood pressure (DBP), height, weight, and waist and hip circumference following the standard protocols. The body mass index (BMI) was calculated as weight in kilograms divided by the square of the height in meters, and the waist-to-hip ratio (WHR) was obtained as waist circumference divided by hip circumference. Blood samples of an overnight fast and 2 h after breakfast were collected for the tests of metabolic indicators, including fasting plasma glucose (FPG), 2-h postprandial plasma glucose (2h PPG), glycosylated hemoglobin A1c (HbA1c), fasting C-peptide (FCP), 2-h postprandial C-peptide (2h PCP), fasting insulin (Fins), 2-h insulin (2hins), total triglycerides (TTG), total cholesterol (TC), high-density lipoprotein cholesterol (HDL-C), low-density lipoprotein cholesterol (LDL-C), lipoprotein a [Lp(a)], alanine aminotransferase (ALT), aspartate aminotransferase (AST), γ-glutamyl transpeptidase (γ-GT), TB, CB, creatinine (Cr), serum uric acid (SUA), and C-reactive protein (CRP), as described in our previous studies (15–19). The 24-h urinary albumin excretion (UAE) was defined as the mean of the values obtained from three separate early-morning urine samples during hospitalization. The homeostasis model assessment index of insulin resistance (HOMA-IR) was calculated as fasting plasma insulin (mU/l) × fasting plasma glucose (mmol/l)/22.5 (20). The homeostasis model assessment for insulin resistance (HOMA2-IR) was determined using the HOMA calculator version 2.2.3, and the estimated glomerular filtration rate (eGFR) was computed according to our previous method (21, 22).

### Carotid ultrasonography

Carotid ultrasonography examinations were performed to evaluate carotid atherosclerotic lesions by trained technicians who were blinded to the clinical data and laboratory findings. That is, three experienced ultrasonographers conducted the carotid ultrasonographic examination using an Acuson Sequoia 512 machine with a 5- to 13-MHz probe following our previously described standard protocol (15, 16, 23). The measurement reproducibilities of carotid atherosclerotic lesions have also been indicated previously (15, 16).

### Diagnostic criteria

The definitions of smoking status, alcohol use, obesity, and hypertension had been well described in our previous studies (15, 24). The definitions of CIMT, carotid plaque, and stenosis had been also described in detail previously by our team (15, 16). Briefly, the CIMT value was calculated as the mean value of the right and left IMTs of the common carotid artery. Carotid plaque was defined by 1) a localized protrusion from the inside of the vessel wall into the lumen at least 0.5 mm or 50% of the surrounding CIMT value and 2) CIMT > 1.5 mm. Carotid stenosis was diagnosed as any degree of narrowing on the carotid arteries caused by plaques.

### Statistical analyses

Statistical analyses were performed using SPSS version 15.0 and R software (version 4.0.2), and figures were made by GraphPad Prism 5.0. For continuous variables, the Kolmogorov–Smirnov test was used to verify the normality of the data. Data with a normal distribution were represented as mean ± S.D., and one-way ANOVA with LSD was used to determine the differences among groups. Data with a skewed distribution were given as median [interquartile range (IQR) 25%–75%], and the Kruskal–Wallis H test was employed to compare the difference among groups. Categorical variables were expressed as absolute numbers (percentages), and the chi-square test was utilized to compare categorical parameters. A restricted cubic spline with four knots (5th, 35th, 65th, and 95th percentiles) was used to detect the dose–response relationships of UCB levels and carotid atherosclerotic lesions after adjusting for age, sex, and DD. Spearman’s correlation analysis was performed to elucidate the interrelationship between UCB and CRP. Binary logistic regression analyses were used to assess the correlations of serum UCB levels and quintiles with carotid atherosclerotic lesions. A value of *p* < 0.05 was considered statistically significant.

## Results

### Characteristics of the study subjects according to UCB quintiles

According to UCB levels, we stratified all subjects into UCB quintiles with cutoffs of <6.2, 6.2–7.9, 8.0–8.9, 9.0–10.9, and >10.9 µmol/l. **Table 1** shows the clinical characteristics of the patients grouped by UCB quintiles. After controlling for age and sex, the subjects in the higher UCB quintiles had remarkably higher levels of DBP, FPG, 2h PPG, HbA1C, TC, HDL-C, LDL-C, ALT, AST, TB, and γ-GT shorter DD. Sex, age, smoking, WHR, 2h C-P, Fins, 2hins, TTG, CB, Cr, SUA, UAE, and eGFR, as well as the use of APAs, LLDs, and IIAs, were also significantly different among the five groups even after adjusting for age and/or sex (all *p* < 0.05).

**Table 1:** Characteristics of the study subjects according to UCB quintiles.

Variables	Q1 (n = 1,553)	Q2 (n = 1,609)	Q3 (n = 1,212)	Q4 (n = 2,014)	Q5 (n = 1,618)	p value	*p value
UCB (µmol/l)	<6.2	6.2-7.9	8.0-8.9	9.0-10.9	>10.9	—	—
Male (n, %)	720 (46.4%)	841 (52.3%)	574 (47.4%)	1074 (53.3%)	944 (58.3%)	<0.001	<0.001
Age (years)	61 ± 12	60 ± 12	61 ± 11	60 ± 11	58 ± 12	<0.001	<0.001
^a^DD (months)	120 (60-180)	108 (48-168)	96 (36-156)	84 (24-135)	72 (12-120)	<0.001	<0.001
Hypertension (n, %)	896 (57.7%)	891 (55.4%)	687 (56.7%)	1073 (53.3%)	833 (51.5%)	0.003	0.578
Obesity (n, %)	711 (45.8%)	727 (45.2%)	573 (47.3%)	914 (45.4%)	743 (45.9%)	0.833	0.786
Smoking (n, %)	432 (27.8%)	474 (29.5%)	337 (27.8%)	574 (28.5%)	491 (30.3%)	0.459	<0.001
Alcohol (n, %)	211 (13.6%)	237 (14.7%)	161 (13.3%)	327 (16.2%)	312 (19.3%)	<0.001	0.456
APAs (n, %)	826 (53.2%)	897 (55.7%)	616 (50.8%)	1011 (50.2%)	754 (46.6%)	<0.001	0.001
AHAs (n, %)	838 (54%)	817 (50.8%)	644 (53.1%)	983 (48.8%)	772 (47.7%)	<0.001	0.294
LLDs (n, %)	650 (41.9%)	694 (43.1%)	470 (38.8%)	730 (36.2%)	614 (37.9%)	<0.001	<0.001
IIAs (n, %)	1147 (73.9%)	1121 (69.7%)	844 (69.6%)	1314 (65.2%)	1077 (66.6%)	<0.001	<0.001
SBP (mmHg)	133 ± 17	133 ± 17	133 ± 18	133 ± 17	132 ± 17	0.770	0.713
DBP (mmHg)	79 ± 9	80 ± 9	80 ± 10	81 ± 9	81 ± 10	<0.001	<0.001
WC (cm)	89.7 ± 10.8	89.9 ± 10.5	89.7 ± 10.4	89.3 ± 10.2	90.2 ± 9.9	0.295	0.470
WHR	0.92 ± 0.07	0.92 ± 0.07	0.92 ± 0.06	0.92 ± 0.06	0.92 ± 0.06	0.016	0.015
BMI (kg/m^2^)	24.89 ± 3.60	24.89 ± 3.45	24.88 ± 3.43	24.78 ± 3.43	24.93 ± 3.41	0.746	0.800
^a^FPG (mmol/l)	7.22 (5.88-9.24)	7.59 (6.11-9.68)	7.61 (6.26-9.59)	7.78 (6.29-9.75)	8.49 (6.73-10.64)	<0.001	<0.001
^a^2h PPG (mmol/l)	12.26 (9.45-15.52)	12.99 (10.06-16.40)	13.11 (9.80-16.12)	13.66 (10.46-16.92)	14.46 (11.17-17.82)	<0.001	<0.001
HbA1C (%)	8.7 ± 2.2	8.9 ± 2.3	8.9 ± 2.2	8.9 ± 2.2	9.1 ± 2.1	<0.001	<0.001
^a^FCP (ng/mL)	1.85 (1.13-2.75)	1.88 (1.13-2.63)	1.76 (1.13-2.59)	1.79 (1.18-2.56)	1.85 (1.24-2.63)	0.436	0.396
^a^2h C-P (ng/mL)	3.99 (2.24-6.03)	3.92 (2.18-6.27)	4.04 (2.22-5.94)	4.19 (2.46-6.23)	4.10 (2.55-6.04)	0.050	0.023
^a^HOMA2-IR	1.56 (0.99-2.33)	1.60 (0.96-2.30)	1.48 (0.96-2.30)	1.57 (1.01-2.20)	1.62 (1.08-2.34)	0.048	0.057
^a^Fins (μU/ml)	12.96 (8.26-22.07)	12.74 (7.70-21.15)	13.35 (8.26-21.02)	12.50 (8.18-19.34)	12.50 (8.17-19.08)	0.023	0.024
^a^2hins (μU/ml)	52.67 (33.76-84.96)	53.29 (34.62-85.45)	53.81 (34.67-81.16)	50.03 (32.20-76.39)	47.42 (30.48-73.79)	<0.001	<0.001
^a^HOMA-IR	4.34 (2.48-8.22)	4.32 (2.55-7.87)	4.67 (2.69-7.85)	4.35 (2.69-7.02)	4.65 (2.95-7.52)	0.114	0.223
^a^TTG (mmol/l)	1.40 (1.00-2.16)	1.44 (0.99-2.14)	1.44 (1.00-2.11)	1.43 (0.99-2.14)	1.61 (1.11-2.31)	<0.001	<0.001
TC (mmol/l)	4.63 ± 1.28	4.76 ± 1.16	4.80 ± 1.06	4.84 ± 1.07	5.07 ± 1.08	<0.001	<0.001
HDL-C (mmol/l)	1.06 ± 0.28	1.11 ± 0.30	1.15 ± 0.30	1.16 ± 0.31	1.18 ± 0.35	<0.001	<0.001
LDL-C (mmol/l)	2.80 ± 0.92	3.00 ± 0.92	3.08 ± 0.90	3.15 ± 0.92	3.30 ± 0.94	<0.001	<0.001
^a^Lp(a)	11.20 (5.90-22.10)	10.70 (5.60-22.38)	10.30 (5.69-20.90)	10.90 (5.90-21.30)	10.51 (5.50-20.60)	0.175	0.064
^a^ALT (U/l)	17 (12-26)	18 (13-27)	19 (13-29)	19 (14-30)	21 (15-33)	<0.001	<0.001
^a^AST (U/l)	18 (15-23)	18 (15-23)	19 (15-24)	19 (16-25)	20 (16-26)	<0.001	<0.001
^a^γ-GT (U/l)	22 (12-26)	23 (16-36)	23 (16-36)	24 (17-38)	26 (18-41)	<0.001	<0.001
^a^TB (µmol/l)	7.4 (6.5-8.3)	9.6 (8.9-10.5)	11 (10-11.9)	12.6 (11.8-13.8)	15.5 (14.3-15.7)	<0.001	<0.001
^a^CB (µmol/l)	2.2 (1.8-3.0)	2.6 (2.0-3.4)	2.5 (2.0-3.5)	3.0 (2.0-4.0)	3.1 (3.0-4.0)	<0.001	<0.001
^a^Cr (μmol/l)	67 (55-83)	66 (55-80)	65 (54-78)	66 (55-78)	66 (55-78)	0.002	<0.001
^a^SUA (μmol/l)	317 (263-383)	314 (261-372)	308 (252-372)	312 (259-373)	311 (259-374)	0.069	0.004
^a^UAE (mg/24 h)	14.61 (7.20-61.11)	12.09 (7.08-37.42)	11.11 (6.75-29.20)	11.29 (6.92-25.34)	10.94 (6.73-26.49)	<0.001	<0.001
^a^eGFR (ml/min/1.73 m^2^)	106.99 (82.08-129.20)	109.16 (88.67-133.55)	110.17 (90.99-132.93)	109.67 (91.65-131.15)	111.64 (94.63-134.83)	<0.001	<0.001

UCB, unconjugated bilirubin; DD, duration of diabetes; APAs, antiplatelet agents; AHAs, antihypertensive agents; LLDs, lipid-lowering drugs; IIAs, insulin or insulin analogues; SBP, systolic blood pressure; DBP, diastolic blood pressure; WC, waist circumstance; WHR, waist–hip ratio; BMI, body mass index; FPG, fasting plasma glucose; 2h PPG, 2-h postprandial plasma glucose; HbA1C, glycosylated hemoglobin A1C; FCP, fasting C-peptide; 2h PCP, 2-h postprandial C-peptide; HOMA-IR, the Homeostasis Model Assessment Indexes-Insulin Resistance; Fins, fasting insulin; 2hins, 2 h insulin; TTG, total triglycerides; TC, total cholesterol; HDL-C, high density lipoprotein-cholesterol; LDL-C, low density lipoprotein-cholesterol; Lp(a), Lipoprotein a; ALT, aspartate aminotransferase; AST, aspartate aminotransferase; γ-GT, γ-glutamyl transpeptidase; TB, total bilirubin; CB, conjugated bilirubin; Cr, creatinine; SUA, serum uric acid; UAE, urinary albumin excretion; eGFR, estimated glomerular filtration rate.

Values are expressed as the mean ± S.D, or median with interquartile range, or percentages. ^a^Non-normal distribution of continuous variables.

p-value: The p-values were not adjusted for age for the trend. *P-value: The *p-values were adjusted for age and sex for the trend.

### Characteristics of serum UCB levels in the study subjects

**Figure 1:**
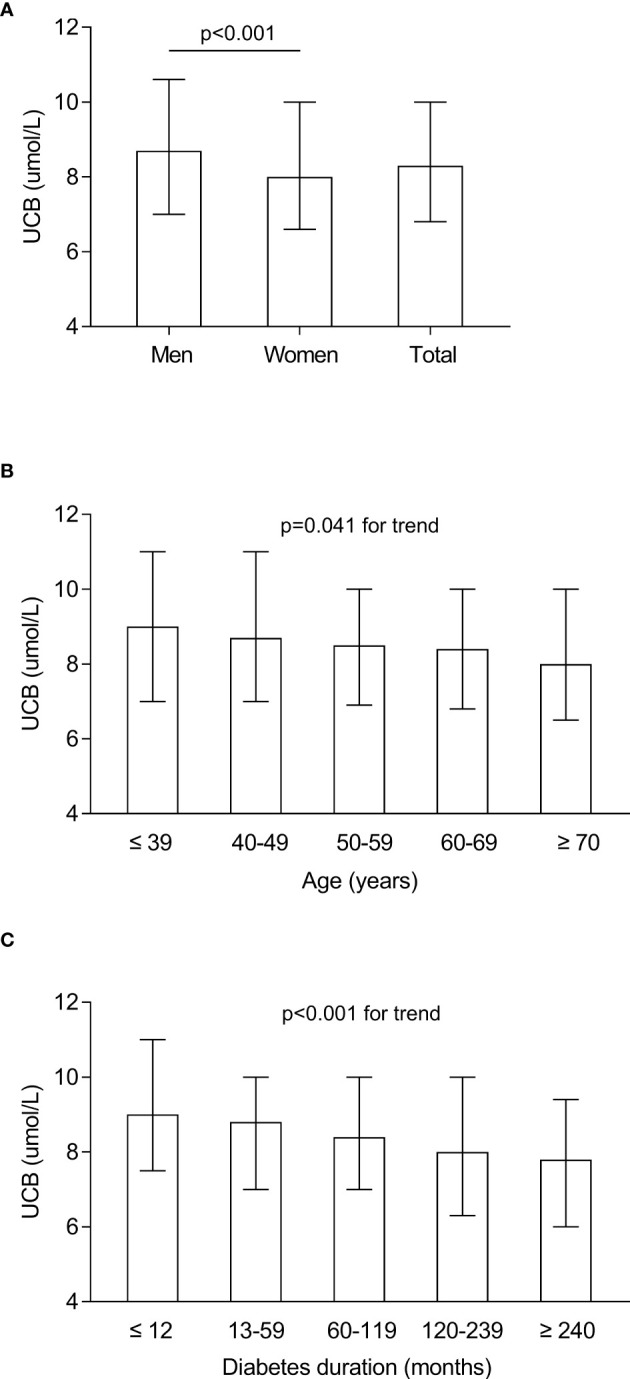
Characteristics of serum UCB levels in the study subjects stratified by sex, age, and DD. **(A)** Comparison of the UCB levels stratified by sex after adjusting for age and DD. **(B)** Comparison of the UCB levels stratified by age after adjusting for sex and DD. **(C)** Comparison of the UCB levels stratified by DD after adjusting for sex and age.

The characteristics of serum UCB levels in the study subjects stratified by sex, age, and DD are presented in **Figure 1**. After controlling for age and DD, a significantly elevated UCB level was found in men compared with women (**Figure 1A**). In addition, significantly decreased trends were observed in the UCB levels along with increased age (*p* = 0.041 for trend) and prolonged DD (*p* < 0.001 for trend) (**Figures 1B, C**).

### Characteristics of carotid atherosclerotic lesions in the study subjects

**Figure 2:**
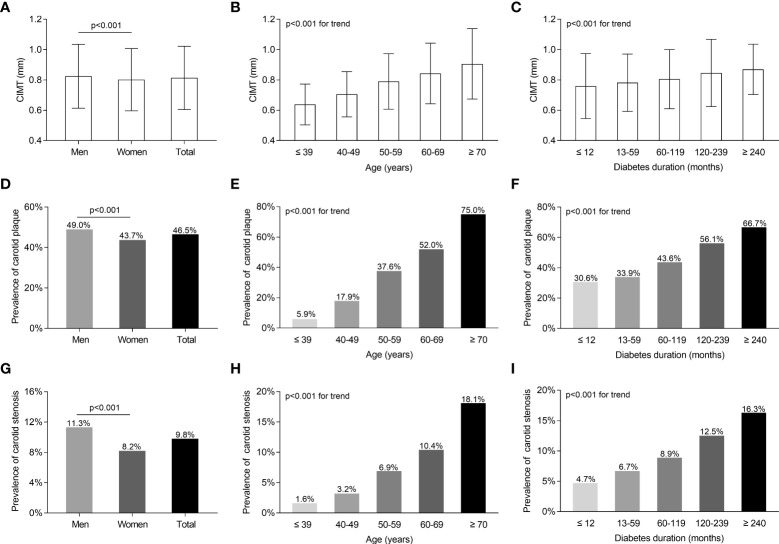
Characteristics of carotid atherosclerotic lesions of the whole subjects with T2DM stratified by sex, age, and DD. **(A)** Comparison of CIMT values stratified by sex after adjusting for age and DD. **(B)** Comparison of CIMT values stratified by age after adjusting for sex and DD. **(C)** Comparison of CIMT values stratified by DD after adjusting for age and sex. **(D)** Comparison of the prevalence of carotid plaque stratified by sex after adjusting for age and DD. **(E)** Comparison of the prevalence of carotid plaque stratified by age after adjusting for sex and DD. **(F)** Comparison of the prevalence of carotid plaque stratified by DD after adjusting for age and sex. **(G)** Comparison of carotid stenosis stratified by sex after adjusting for age and DD. **(H)** Comparison of the prevalence of carotid stenosis stratified by age after adjusting for sex and DD. **(I)** Comparison of carotid stenosis stratified by DD after adjusting for age and sex.

The characteristics of carotid atherosclerotic lesions in all participants stratified by sex, age, and DD are shown in **Figure 2**. Male patients had a notably greater CIMT value (0.82 ± 0.21 mm *vs*. 0.80 ± 0.21 mm), a higher prevalence of carotid plaque (49% *vs*. 43.7%), and stenosis (11.3% *vs*. 8.2%) compared with female patients after controlling for age and DD (**Figures 2A, D, G**). After adjusting for sex and DD, the CIMT value and the prevalence of carotid plaque and stenosis were successively found to remarkably rise with age and DD (**Figures 2B, E, H, C, F** **, I**).

### Comparisons of carotid atherosclerotic lesions among the UCB quintile groups

**Figure 3:**
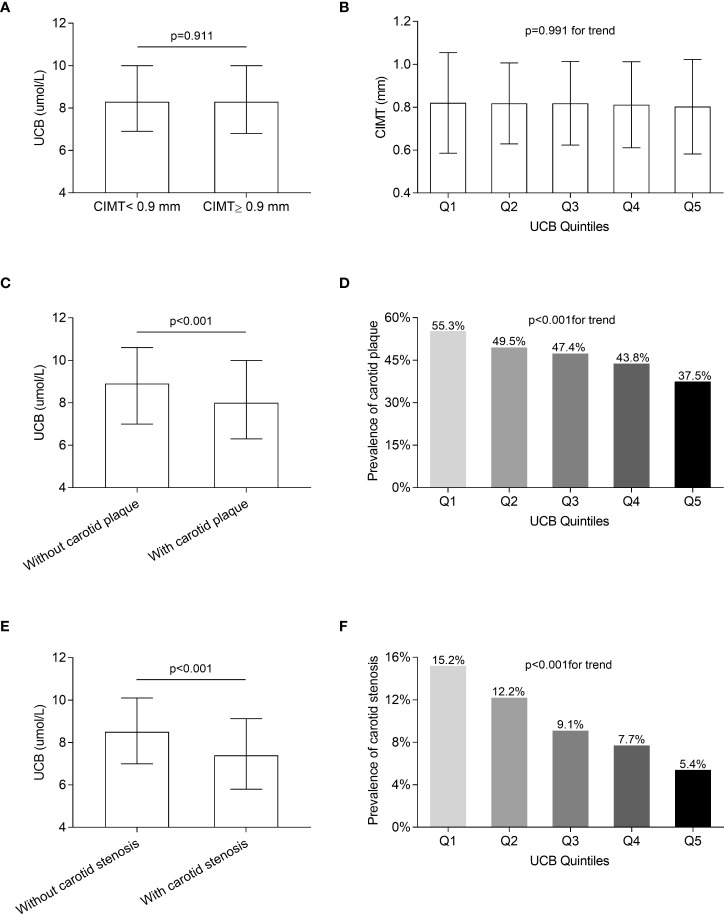
Comparison of carotid atherosclerotic lesions among the UCB quintile groups. **(A)** Comparison of UCB levels between the subjects with CIMT ≥0.9 mm and <0.9 mm after controlling for age, sex, and DD. **(B)** Comparison of the CIMT value among the UCB quintile groups after adjusting for age, sex, and DD. **(C)** Comparison of UCB levels between the subjects with and without carotid plaque after controlling for age, sex, and DD. **(D)** Comparison of the prevalence of carotid plaque among the UCB quintile groups after adjusting for age, sex, and DD. **(E)** Comparison of UCB levels between the subjects with and without carotid stenosis after controlling for age, sex, and DD. **(F)** Comparison of the prevalence of carotid stenosis among the UCB quintile groups after adjusting for age, sex, and DD.

**Figure 4:**
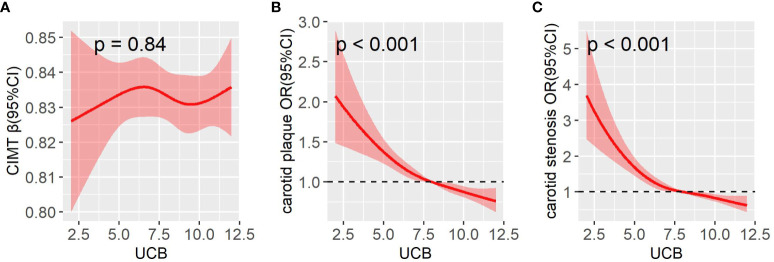
A restricted cubic spline (RCS) was used to detect the dose–response relationship of UCB with CIMT **(A)**, carotid plaque **(B)**, and carotid stenosis **(C)** after adjusting for age, sex, and DD. The solid line and the shaded portion represented the estimated odds ratio and its 95% CI. Knots are at the 5th, 35th, 65th, and 95th percentiles for UCB. CI, confidence interval.

**Figure 3** displays the comparisons of carotid atherosclerotic lesions including CIMT, carotid plaque, and stenosis across the UCB quintile groups. A remarkable decrease in the prevalence of carotid plaque and stenosis was observed across the UCB quintiles (carotid plaque: 55.3%, 49.5%, 47.4%, 43.8%, and 37.5%, respectively, p < 0.001 for trend; carotid stenosis:15.2%, 12.2%, 9.1%, 7.7%, and 5.4%, respectively, p < 0.001 for trend) (**Figure 3D, F**). Moreover, the patients with carotid plaque showed a significantly lower serum UCB concentration than those without carotid plaque [8 (IQR 6.3–10) µmol/l *vs*. 8.9 (IQR 7–10.6) µmol/l, *p* < 0.001] (**Figure 3C**). Similarly, the patients with carotid stenosis also had obviously lower UCB levels than those without carotid stenosis [7.4 (IQR 5.8–9.1) µmol/l *vs*. 8.5 (IQR 7–10.1) µmol/l, *p* < 0.001] (**Figure 3E**). However, there was no significant difference in the CIMT value across the UCB quintile groups (**Figure 3B**). Additionally, there was no obvious difference in UCB levels between the patients with CIMT ≥0.9 mm and CIMT <0.9 mm [8.3 (IQR 6.8–10.0) µmol/l *vs*. 8.3 (IQR 6.9–10.0) µmol/l, *p* = 0.911] (**Figure 3A**). In addition, as shown in **Figure 4**, a non-linear inverse dose–response was observed between UCB concentration and carotid plaque as well as stenosis, but not CIMT after adjusting for age, sex, and DD (**Figures 4A** **–C**).

### Association of UCB with CRP

**Figure 5:**
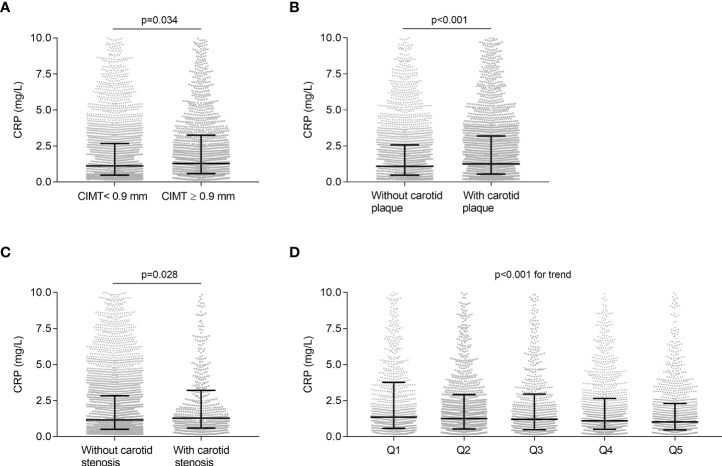
Association of UCB with CRP. **(A)** Comparison of CRP levels between the subjects with CIMT <0.9 mm and with ≥0.9 mm after controlling for age, sex, and DD. **(B)** Comparison of CRP levels between the subjects with and without carotid plaque after controlling for age, sex, and DD. **(C)** Comparison of CRP levels between the subjects with and without carotid stenosis after controlling for age, sex, and DD. **(D)** Comparison of CRP levels among the UCB quintile groups. Data are shown as the median with 25th and 75th percentiles.

Spearman’s correlation analysis was performed to explore the association between UCB levels and CRP, and the results revealed that serum UCB levels were negatively associated with CRP (r = -0.088, *p* < 0.001) even after adjusting for age, sex, and DD. In addition, **Figure 5** illustrates the comparison of CRP in different groups. The levels of CRP were significantly increased in the patients with CIMT ≥0.9 mm relative to those with CIMT <0.9 mm (p = 0.034, **Figure 5A**), and the same was found in carotid plaque (*p* < 0.001, **Figure 5B**) and stenosis (*p* = 0.028, **Figure 5C**). Moreover, a significant decrease in CRP levels was observed from the lowest to highest UCB quintiles (*p* < 0.001 for trend, **Figure 5D**).

### Associations of UCB levels and quintiles with carotid plaque and stenosis

**Table 2** demonstrates the associations of serum UCB quintiles with carotid plaque and stenosis. The remarkably inverse associations of UCB quintiles with carotid plaque and stenosis were found in model 1. After further controlling for other potential confounders (models 2–5), there were still significantly negative correlations between UCB quintiles and the presence of carotid plaque and stenosis. Accordingly, we found that the risk of carotid plaque decreased by 25.5%, 28.7%, 33.5%, and 42.8%, and that of carotid stenosis by 24.6%, 37.4%, 44.9%, and 47.3%, respectively, in those from the second to highest UCB quintiles when compared with the subjects in the first UCB quintile.

**Table 2:** Associations of serum UCB quintiles with carotid plaque and stenosis.

	ORs (95% CI)					p valuefor trend
	Q1	Q2	Q3	Q4	Q5	
**Carotid plaque**						
Model 1	1 (ref)	0.793 (0.689-0.912)	0.727 (0.625-0.845)	0.631 (0.552-0.721)	0.484 (0.420-0.558)	<0.001
Model 2	1 (ref)	0.807 (0.689-0.945)	0.733 (0.619-0.869)	0.669 (0.575-0.777)	0.558 (0.475-0.655)	<0.001
Model 3	1 (ref)	0.736 (0.621-0.871)	0.725 (0.605-0.869)	0.656 (0.559-0.771)	0.543 (0.458-0.645)	<0.001
Model 4	1 (ref)	0.757 (0.632-0.907)	0.717 (0.588-0.875)	0.679 (0.569-0.811)	0.581 (0.478-0.705)	<0.001
Model 5	1 (ref)	0.745 (0.612-0.906)	0.713 (0.573-0.887)	0.665 (0.546-0.809)	0.572 (0.460-0.711)	<0.001
**Carotid stenosis**						
Model 1	1 (ref)	0.774 (0.631-0.949)	0.557 (0.438-0.708)	0.469 (0.378-0.580)	0.321 (0.249-0.414)	<0.001
Model 2	1 (ref)	0.800 (0.648-0.988)	0.580 (0.453-0.743)	0.519 (0.415-0.647)	0.379 (0.292-0.494)	<0.001
Model 3	1 (ref)	0.763 (0.615-0.947)	0.585 (0.455-0.753)	0.532 (0.424-0.667)	0.389 (0.298-0.508)	<0.001
Model 4	1 (ref)	0.759 (0.607-0.949)	0.604 (0.463-0.788)	0.590 (0.466-0.747)	0.473 (0.358-0.627)	<0.001
Model 5	1 (ref)	0.754 (0.590-0.964)	0.626 (0.468-0.839)	0.551 (0.422-0.720)	0.527 (0.385-0.722)	<0.001

Model 1: unadjusted.

Model 2: age, sex, DD, smoking status, alcohol intake, hypertension, and obesity.

Model 3: model 2 + use of APAs, AHAs, LLDs and IIAs.

Model 4: model 3 + SBP, DBP, WC, WHR and BMI.

Model 5: model 4 + ALT, AST, γ-GT, TTG, TC, HDL-C, LDL-C, Lp(a), eGFR, SUA, UAE, FPG, 2h PPG, HbA1C, FCP, 2h PCP, Fins, 2hins, and CRP.

**Table 3** presents the associations of serum UCB levels with carotid plaque and stenosis. We found that serum UCB levels within the normal limits were negatively correlated with carotid plaque and stenosis in the unadjusted model. Further controlling for various clinical indicators (models 2–5), decreased serum UCB levels remained as an independent risk factor for carotid plaque [odds ratio (OR) 0.810, 95% confidence interval (CI) 0.747–0.878; *p* < 0.001] and stenosis [OR 0.722, 95%CI 0.647-0.805; *p* < 0.001].

**Table 3:** Associations of serum UCB levels with carotid plaque and stenosis.

		β	S.E.	Wald	p value	OR	95% CI for OR	
							Lower	Upper
**Carotid plaque**								
Model 1	1 (ref)	-0.285	0.027	110.227	<0.001	0.752	0.713	0.793
Model 2	1 (ref)	-0.232	0.031	56.595	<0.001	0.793	0.746	0.842
Model 3	1 (ref)	-0.235	0.033	51.093	<0.001	0.791	0.741	0.843
Model 4	1 (ref)	-0.216	0.036	35.262	<0.001	0.806	0.750	0.865
Model 5	1 (ref)	-0.211	0.041	26.257	<0.001	0.810	0.747	0.878
**Carotid stenosis**								
Model 1	1 (ref)	-0.474	0.043	119.069	<0.001	0.623	0.572	0.678
Model 2	1 (ref)	-0.418	0.046	83.325	<0.001	0.658	0.602	0.720
Model 3	1 (ref)	-0.404	0.047	75.158	<0.001	0.668	0.609	0.732
Model 4	1 (ref)	-0.336	0.049	47.439	<0.001	0.714	0.649	0.786
Model 5	1 (ref)	-0.325	0.056	34.144	<0.001	0.722	0.647	0.805

Model 1: unadjusted.

Model 2: age, sex, DD, smoking status, alcohol intake, hypertension, and obesity.

Model 3: model 2 + use of APAs, AHAs, LLDs, and IIAs.

Model 4: model 3 + SBP, DBP, WC, WHR, and BMI.

Model 5: model 4 + ALT, AST, γ-GT, TTG, TC, HDL-C, LDL-C, Lp(a), eGFR, SUA, UAE, FPG, 2h PPG, HbA1C, FCP, 2h PCP, Fins, 2hins, and CRP.

## Discussion

In this cross-sectional, real-world study, the impact of serum UCB within the normal limits on carotid atherosclerotic lesions was assessed in Chinese T2DM patients. Our results clearly demonstrated that higher serum UCB within the normal range had a remarkably inverse association with late carotid atherosclerotic lesions including carotid plaque and stenosis but not with CIMT, an early carotid atherosclerotic lesion in T2DM patients, even after adjusting for various confounders. Moreover, high-normal UCB may be protective against carotid atherosclerotic lesions by an anti-inflammation effect, which is indicated by the decreased CRP levels from low to high UCB quintiles.

Although the association between serum bilirubin and atherosclerotic cardiovascular diseases has been extensively studied, data on T2DM patients are sparse and conflicting. Inoguchi et al. (25) firstly found that diabetic patients with Gilbert syndrome, a common hereditary genetic disorder causing hyperbilirubinemia, faced a lower risk of coronary artery disease and cerebrovascular disease (CBVD) than those with normal bilirubin concentrations. In 674 Japanese T2DM patients, Nishimura et al. (26) also displayed that the patients with CBVD or peripheral arterial disease (PAD) had successively lower TB concentrations than those without these vascular complications. In contrast, Yeh et al. (27) found that TB seemed to have no beneficial effect on macrocirculation identified by flow-mediated dilation and nitroglycerin-induced dilation in diabetic patients, including 37 type 1 diabetes and 213 type 2 diabetes. The paradoxical conclusions may be related to the exposure levels and types of bilirubin in the studied objects. For example, a U-shaped relationship between bilirubin and CHD was reported; that is, TB levels within the range of 12–16 µmol/l had the strongest protective effect on CHD (9, 28, 29). Furthermore, Chen and colleagues (30) analyzed the associations of different types of bilirubin (D-BIL, I-BIL, and TB) with diabetic foot (DF) separately and discovered that serum I-BIL, but not TB and D-BIL, played a protective role in DF, and I-BIL and TB but not D-BIL were independent risk factors for the severity of DF. The findings of Chen et al. (30) indicated that D-BIL may be highly effective in anti-atherosclerosis activity, which was also confirmed by *in vivo* and *in vitro* experiments in which UCB exerted major antioxidant properties by virtue of its hydrophobicity (13, 14, 31). Furthermore, to the best of our knowledge, few studies have analyzed the potential values of serum bilirubin within physiological concentrations in atherosclerosis. Therefore, whether serum UCB within the normal limits had some connections with carotid atherosclerotic lesions in T2DM patients is worth exploring.

Up to now, only a few studies have made the comparisons on CIMT values between low and high bilirubin concentration groups. In 111 healthy men without manifestations of atherosclerosis, Vítek et al. (32) found that the mean CIMT of men with hyperbilirubinemia was substantially lower than that of men with normobilirubinemia. Additionally, Erdogan et al. (33) demonstrated that the subjects with serum TB levels in the lower third of the reference range had significantly higher CIMT values than those with serum TB levels in the upper third of the reference range among 91 healthy participants. Furthermore, Dullaart et al. (34) also discovered that CIMT negatively correlated with TB concentrations in 80 T2DM subjects without clinical manifestations of cardiovascular disease. Different from the abovementioned studies, we did not find a remarkable difference between CIMT and serum UCB within the physiologic range in a large sample of 8,006 T2DM patients when the subjects were divided into quintiles by serum UCB levels.

In addition, some observational studies illustrated that serum TB levels were independently correlated with subclinical atherosclerosis (CIMT values <0.9 *vs*. ≥0.9 mm) in various individuals like prehypertensive patients (35) and prediabetic patients (36). However, the range of serum TB values was not limited, and UCB was not included in their studies. Recently, among 84 patients with obstructive sleep apnea, Duman et al. (37) found that the patients with CIMT ≥0.9 mm had significantly lower TB and I-BIL levels than those with CIMT <0.9 mm, but only TB (OR 0.72, 95% CI 0.60–0.86, *p* < 0.01) and not I-BIL (OR 1.11, 95% CI 0.73–1.69, *p* = 0.61) was the independent predictor of CIMT ≥0.9 mm. Our study found that no marked difference on the UCB levels was observed between T2DM patients with CIMT ≥0.9 mm and <0.9 mm. Different types of bilirubin involved in the studies, different studied populations, race, and sample sizes may help to explain the inconsistent results regarding bilirubin and CIMT. For example, based on a relatively large sample, the subjects in our study were diabetic patients with an average age of about 60 years who were at high risk of atherosclerosis. Moreover, large-scale prospective cohorts may need to further verify our findings.

In our study, we comprehensively assessed the effect of UCB within the physiological range on carotid atherosclerotic lesions including CIMT, carotid plaque, and stenosis and found that the prevalence of carotid plaque and stenosis was progressively lowered with the increased UCB quintiles. The results of fully adjusted regression analyses also showed that the elevated UCB level, both as quintiles and as a continuous variable, was independently and negatively associated with carotid plaque and stenosis. However, no remarkable association was observed between serum UCB and CIMT in T2DM patients. Our present findings indicated that a high serum UCB level, even within the normal range, could protect T2DM patients from late but not early carotid atherosclerotic lesions. To the best of our knowledge, it is the first time that the relations of serum UCB and carotid atherosclerotic lesions in patients with T2DM were comprehensively explored.

Data on serum bilirubin and carotid plaque and stenosis were very limited and primarily concentrated on serum TB. Similar with us, Ishizaki et al. (38) discovered that 330 subjects (19%) were diagnosed to have carotid plaque in either or both of carotid arteries, and the odds ratio of carotid plaque was 0.37 with the increase in serum TB concentration by 1.0 mg/dl in 1,741 general subjects who underwent health-screening tests. Kawamoto et al. (39) also demonstrated that a mildly elevated serum TB level was independently and negatively related to carotid plaque in elderly persons aged >60 years, independent of other confounding factors. By contrast, a community-based study involving 245 healthy individuals revealed that no independent relationship existed between TB at baseline and carotid plaque at both baseline and follow-up (40). This difference may be attributed to the bilirubin exposure levels and the different basic characteristics. Aligned with them, Lapenna and colleagues (13) showed that the levels of serum bilirubin, especially I-BIL, were remarkably decreased in the subjects with a carotid stenosis degree greater than 90% compared with those with a carotid stenosis degree less than 90% in 32 patients scheduled for elective carotid artery endarterectomy.

Although the impact of UCB within the physiological range on carotid atherosclerosis has yet to be clear, several reasons may help to explain the association between UCB and carotid atherosclerotic lesions. Firstly, an elevated UCB level might physiologically exert antioxidant and anti-lipoperoxidative effects by suppressing the propagation of lipid peroxidation and scavenging against peroxyl radicals (14, 31, 41). Secondly, a higher UCB level may also have an anti-inflammatory effect on the development of atherosclerosis. CRP has been known as a sensitive marker of chronic systemic inflammation such as atherosclerosis (42, 43). Yoshino et al. (44) demonstrated that serum TB was negatively correlated with CRP, and CRP could independently predict the TB level in overweight patients. A retrospective cross-sectional study also found that the TB level was negatively associated with inflammatory markers including CRP in patients with coronary atherosclerosis (45). Our study further displayed that CRP levels were remarkably lower in subjects with carotid atherosclerosis compared with those without carotid atherosclerosis and clearly decreased across the serum UCB quintiles. These results suggested that high-normal UCB levels could reduce pro-inflammatory cytokines and might also inhibit the inherent inflammatory process of carotid atherosclerosis. Thirdly, UCB was negatively associated with carotid plaque and stenosis, but not with CIMT, which may stem from the dissimilar pathological processes of atherosclerotic lesions. That is, the biggest difference is that CIMT measurements are routinely obtained in the common carotid artery, whereas plaque formation and progression predominantly occur downstream of the internal carotid (46). Additionally, age and hypertension were also the primary contributors to IMT (47), which did not necessarily reflect the process of atherosclerosis. Moreover, carotid plaques were initiated by pathological intimal thickening and characterized by the formation of lipid pools in the absence of a necrotic core (48). In addition, UCB might also take part in preventing atherosclerosis by exhibiting anticomplement activity and attenuating vascular endothelial activation and dysfunction (49, 50).

Some limitations should be mentioned. Firstly, the present study mainly covered middle-aged and elderly Chinese T2DM subjects, and further studies may need to verify whether our findings could be generalized to younger Asians or other ethnic groups. Secondly, the patients with elevated serum bilirubin levels were excluded from our study, but it is not certain whether patients with Gilbert syndrome had been completely eliminated in our study due to its high prevalence (4%–16%) for people in China (51). Thirdly, our study was a cross-sectional study, and further studies may need to help establish the causal association between high-normal UCB levels and carotid atherosclerotic lesions. Therefore, further prospective studies are needed to replicate and confirm our findings.

## Conclusions

In conclusion, the present study demonstrated that elevated serum UCB levels within normal limits were independently and inversely correlated with the presence of late carotid atherosclerotic lesions including carotid plaque and stenosis but not with CIMT, an early carotid atherosclerotic lesion in T2DM patients. Furthermore, high-normal UCB levels may play a protective role in the late carotid atherosclerotic process through an anti-inflammation effect, which can be indicated by the decreased CPR levels from low to high UCB quintiles. In clinical practice, as a simple and readily available biomarker of cardiovascular risk, serum UCB may be used as a protective indicator to evaluate the risk of atherosclerosis in T2DM patients.

## Data availability statement

The original contributions presented in the study are included in the article/supplementary material. Further inquiries can be directed to the corresponding authors.

## Ethics statement

The studies involving human participants were reviewed and approved by Ethics Committee of Shanghai Jiao Tong University Affiliated Sixth People’s Hospital. The patients/participants provided their written informed consent to participate in this study.

## Author contributions

M-FL and L-XL designed the study and reviewed and edited the manuscript. J-WW, J-FK and J-BL collected samples and clinical data. C-HJ and J-WW worked together, performed the statistical analysis, and wrote the manuscript. All authors contributed to the article and approved the submitted version.

## Funding

This work was supported by the National Natural Science Foundation of China (grant numbers 81170759, 81770813, 82070866, and 81502316), the National Key Research and Development Plan (grant numbers 2018YFC1314900 and 2018YFC1314905), the Translational Medicine National Key Science and Technology Infrastructure Open Project (grant number TMSK-2021-116), the Exploratory Clinical Research Project of Shanghai Jiao Tong University Affiliated Sixth People’s Hospital (grant number ynts202105), and Shanghai Municipal Key Clinical Specialty. The funders were not involved in the study design, collection, analysis, interpretation of data, the writing of this article, or the decision to submit it for publication.

## Conflict of interest

The authors declare that the research was conducted in the absence of any commercial or financial relationships that could be construed as a potential conflict of interest.

## Publisher’s note

All claims expressed in this article are solely those of the authors and do not necessarily represent those of their affiliated organizations, or those of the publisher, the editors and the reviewers. Any product that may be evaluated in this article, or claim that may be made by its manufacturer, is not guaranteed or endorsed by the publisher.
